# Supplementary material for: Content-rich biological network constructed by mining PubMed abstracts
Source: BMC Bioinformatics. 2004 Oct 8;5:147. doi: 10.1186/1471-2105-5-147 (PMC528731; doi:10.1186/1471-2105-5-147)
Supplement: Additional File 2 — The original results of the above study (non-essential files are deleted to keep the file size under the limit set by BMC bioinformatics). [file 1471-2105-5-147-S2.bz2 › chilibotAdditionalFile2/dip50/249ID7760852E1003/html/RARA_RXRA.html]

 


 **RARA** and **RXRA** 
  
Found 92 abstracts in PubMed, retrieved 50.  
 

 What does Google say? 
 PDF only 
| .edu only 

---

**Interactive relationship** (e.g. stimulation, inhibition, etc)

**Neutral relationship**- The recent discovery of the interaction between nuclear IGFBP 3 and 9 cis retinoic acid receptor alpha  [ **RARA** ]  retinoid X receptor alpha  [ **RXRA** ]  RXRalpha, a nuclear receptor, and its involvement in the regulation of transcriptional signaling and apoptosis represents an important paradigm shift in the understanding of IGFBP function.  Ref: 12379488 J Endocrinol, 2002
- The RA receptor alpha RAR alpha  [ **RARA** ]  isoform bound to the oligomer representing the sequences between 125 and 100 as a heterodimer complex with the retinoid X receptor alpha  [ **RXRA** ]  RXR alpha.  Ref: 9448745 Biochem Pharmacol, 1998
- Both the human retinoic acid receptor alpha  [ **RARA** ]  hRARalpha and a constitutively active mutant F318A of the mouse retinoid X receptor alpha  [ **RXRA** ]  mRXR alpha F318A ligand binding domains were separately overexpressed in Escherichia coli, copurified as a heterodimer in a two step procedure, and cocrystallized with an RAR alpha  [ **RARA** ]  specific antagonist by using polyethylene glycol 1 00 as precipitant.  Ref: 10873543 Protein Expr Purif, 2000

**Non-interactive relationship** (e.g. studied together, co-existance, homology, etc.)

- Increased retinoic acid responsiveness in lung carcinoma cells that are nonresponsive despite the presence of endogenous retinoic acid receptor RAR  [ **RARA** ]  beta by expression of exogenous retinoid receptors retinoid X receptor alpha  [ **RXRA** ] , RAR alpha  [ **RARA** ] , and RAR  [ **RARA** ]  gamma.  Ref: 11212249 Cancer Res, 2001
- Moreover, DMS induced the expression of retinoic acid receptor alpha  [ **RARA** ]  and retinoid X receptor alpha  [ **RXRA** ] .  Ref: 11520048 Biochem Biophys Res Commun, 2001
- Reverse transcription polymerase chain reaction analysis showed that the retinoic acid receptors RAR alpha  [ **RARA** ] , RAR  [ **RARA** ]  gamma, and retinoid X receptor alpha  [ **RXRA** ]  were present in the cells.  Ref: 11678760 BJU Int, 2001
- Retinoid receptor expression was evaluated by reverse transcriptase polymerase chain reaction RT PCR using subtype specific primers and demonstrated expression of retinoic acid receptor alpha  [ **RARA** ]  RAR alpha  [ **RARA** ] , RAR  [ **RARA** ]  beta and retinoid X receptor alpha  [ **RXRA** ]  RXR alpha.  Ref: 9823968 Br J Cancer, 1998
- NIX1 selectively binds retinoic acid receptor RAR  [ **RARA** ]  NR1A and thyroid hormone receptor TR NR1B  [ **RARA** ]  in a ligand dependent manner, but does not interact with retinoid X receptor RXR NR2B  [ **RXRA** ]  or steroid hormone receptors.  Ref: 10860982 Proc Natl Acad Sci U S A, 2000
- Whole cell voltage clamp recordings were performed to assess the ability of conantokin G, conantokin T, and a 17 residue truncated form of conantokin R to inhibit N methyl d aspartate evoked currents in human embryonic kidney 293 cells transiently expressing various combinations of NR1a, NR1b  [ **RARA** ] , NR2A, and NR2B  [ **RXRA** ]  receptor subunits.  Ref: 11335724 J Biol Chem, 2001
- PP1c specifically interacts with VDR but not retinoic acid receptor alpha  [ **RARA** ]  and retinoid X receptor alpha  [ **RXRA** ]  in yeast.  Ref: 12036952 J Biol Chem, 2002
- Bexarotene treatment suppressed the expression of retinoid X receptor alpha  [ **RXRA** ]  and retinoic acid receptor alpha  [ **RARA** ]  proteins in all three lines compared with untreated controls.  Ref: 12006543 Clin Cancer Res, 2002
- Although retinoic acid receptor RAR alpha  [ **RARA** ] , beta, gamma and retinoid X receptor alpha  [ **RXRA** ]  were expressed in all cell lines examined, a significant induction of RARbeta by all trans RA was observed only in sensitive cell lines, suggesting important roles of RARbeta in RA sensitivity.  Ref: 10660115 Biochem Pharmacol, 2000
- The pH sensitivity of ifenprodil block occurs at NR1B  [ **RARA** ]  NR2B  [ **RXRA** ]  as well as NR1A NR2B  [ **RXRA** ]  receptors, suggesting that it is not influenced by the exon 5 insert that is present in NR1B  [ **RARA** ]  but absent in NR1A.  Ref: 9143010 Neurosci Lett, 1997
- Sequence comparisons and phylogenetic analyses reveal that these ascidian nuclear receptors are representative of five of the six previously defined nuclear receptor subfamilies and are apparent homologues of retinoic acid NR1B  [ **RARA** ] , retinoid X NR2B  [ **RXRA** ] , peroxisome proliferator activated NR1C, estrogen related NR3B, neuron derived orphan NOR NR4A3, nuclear orphan NR4A, TR2 orphan NR2C1 and COUP orphan NR2F3 receptors.  Ref: 12141458 Int J Dev Biol, 2002
- We show here that a mutant TR beta corrected identified in a patient with RTH inhibited the transcriptional activities of, not only the wild type TR beta, but also other nuclear receptors including retinoid X receptor alpha  [ **RXRA** ]  RXR alpha, vitamin D3 receptor VDR and retinoic acid receptor RAR alpha  [ **RARA** ] .  Ref: 9299447 Biochem Biophys Res Commun, 1997
- Retinoic acid modulates retinoid X receptor alpha  [ **RXRA** ]  and retinoic acid receptor alpha  [ **RARA** ]  levels of cultured brown adipocytes.  Ref: 9109417 FEBS Lett, 1997
- The full length GRIP1 interacted with the hormone binding domains HBDs of all five steroid receptors in a hormone dependent manner and also with HBDs of class II nuclear receptors, including thyroid receptor alpha, vitamin D receptor, retinoic acid receptor alpha  [ **RARA** ] , and retinoid X receptor alpha  [ **RXRA** ] .  Ref: 9111344 Mol Cell Biol, 1997
- The two nuclear receptor binding motifs within the PBREM effectively bound PXR as a heterodimer with the 9 cis retinoic acid receptor alpha  [ **RARA** ]  NR2B1  [ **RXRA** ] .  Ref: 11502872 Mol Pharmacol, 2001
- By analyzing the effects of Zn2 on varied combinations of NR1 NR1a or NR1b  [ **RARA** ]  and NR2 NR2A, NR2B  [ **RXRA** ] , NR2C, we show that both the NR1 and the NR2 subunits contribute to the voltage independent Zn2 inhibition.  Ref: 9221770 J Neurosci, 1997
- A number of aminoglycosides were found to potentiate macroscopic currents at heteromeric NR1A NR2B  [ **RXRA** ]  receptors, but not at NR1A NR2A, NR1A NR2C, NR1A NR2D, or NR1B  [ **RARA** ]  NR2B  [ **RXRA** ]  receptors.  Ref: 10454474 J Pharmacol Exp Ther, 1999
- We wished to determine the effect of altering the levels or functional activity of retinoid receptors, in particular retinoic acid receptor alpha  [ **RARA** ]  RAR alpha  [ **RARA** ]  and retinoid X receptor alpha  [ **RXRA** ]  RXR alpha on the growth sensitivity of ovarian tumor cells to all trans retinoic acid all trans RA.  Ref: 9417874 Exp Cell Res, 1997
- Three of 10 compounds displayed significantly higher affinities for NR1b  [ **RARA** ]  NR2C receptors than NR1b  [ **RARA** ]  NR2A receptors, and three of five compounds had higher affinity at NR1b  [ **RARA** ]  NR2C than NR1b  [ **RARA** ]  NR2B  [ **RXRA** ]  receptors.  Ref: 9023271 J Pharmacol Exp Ther, 1997
- Here we provide evidence that the UCP responsiveness to RA in primary cultures of brown adipocytes involves RA receptor alpha RAR alpha  [ **RARA** ] , and show, in the same system and also in CHO cells, that RA down regulates the steady state levels of RAR alpha  [ **RARA** ]  and especially of retinoid X receptor alpha  [ **RXRA** ] , suggesting autoregulation of the retinoid pathway and therefore supporting the idea of a physiological role for it in controlling the thermogenic capacity of BAT.  Ref: 9109417 FEBS Lett, 1997
- CONCLUSIONS Bexarotene treatment at clinically relevant concentrations causes apoptosis of CTCL cell lines in association with activation of caspase 3 and cleavage of poly ADP Ribose polymerase, as well as down regulation of retinoid X receptor alpha  [ **RXRA** ] , retinoic acid receptor alpha  [ **RARA** ] , and survivin.  Ref: 12006543 Clin Cancer Res, 2002
- Furthermore, prolongation of NR1a NR2A responses was similar to that of responses from NR1b  [ **RARA** ]  NR2B  [ **RXRA** ]  receptor, a subtype characterized by reduced desensitization.  Ref: 11249958 Neuropharmacology, 2001
- NR1b  [ **RARA** ]  NR2B  [ **RXRA** ]  and NR1b  [ **RARA** ]  NR2A were distinguished by one of five compounds tested.  Ref: 9023271 J Pharmacol Exp Ther, 1997
- These results, differential proteolysis sensitivity assays, and glutathione S transferase pulldown experiments demonstrate that, unlike AHPN or the natural or standard synthetic retinoids, MM11453 does not behave as a RAR  [ **RARA** ]  or retinoid X receptor alpha  [ **RXRA** ]  transcriptional agonist.  Ref: 11406543 Cancer Res, 2001
- TRAP220 also interacts with other nuclear receptors vitamin D receptor, retinoic acid receptor alpha  [ **RARA** ] , retinoid X receptor alpha  [ **RXRA** ] , peroxisome proliferation activated receptor PPAR alpha, PPARgamma and, to a lesser extent, estrogen receptor in a ligand dependent manner.  Ref: 9653119 Proc Natl Acad Sci U S A, 1998
- In this study, we examined the binding characteristics of the nuclear extract of HSG cells to the retinoic acid response element RARE compared with those of in vitro translated RAR alpha  [ **RARA** ]  and retinoid X receptor alpha  [ **RXRA** ]  RXR alpha, a heterodimeric partner of RAR alpha  [ **RARA** ] .  Ref: 9599664 Biochem Cell Biol, 1997
- Cell morphology, total culture protein content, the DNA synthesis rate, and the levels of UCP1, retinoic acid receptor alpha  [ **RARA** ]  RARalpha and retinoid X receptor alpha  [ **RXRA** ]  RXRalpha were analysed.  Ref: 10411240 Int J Obes Relat Metab Disord, 1999
